# Effect of food intake on respiratory chemosensitivity to CO_2_ in young adults

**DOI:** 10.1186/s40101-019-0200-7

**Published:** 2019-07-08

**Authors:** Keiji Hayashi, Misato Suekuni, Koji Sugiyama

**Affiliations:** 1 Junior College, University of Shizuoka, 2-2-1 Oshika, Suruga-ku, Shizuoka, 422-8021 Japan; 2 Faculty of Education, Shizuoka University, Shizuoka, Japan

**Keywords:** Respiratory chemoreflex, Ventilation, Hypercapnia

## Abstract

**Background:**

Food intake augments CO_2_ production; however, minute ventilation is not augmented during exercise after food intake. Respiratory chemoreceptors respond to CO_2_ and influence respiration. We examined the effect of food intake on respiratory chemosensitivity to CO_2_ in young adults.

**Methods:**

The hypercapnic ventilatory response was measured in eleven healthy individuals before and after food intake. To evaluate the respiratory chemoreflex response to CO_2_, minute ventilation was plotted against end-tidal PCO_2_ using data obtained with the rebreathing method.

**Results:**

Sublingual temperature, CO_2_ output, minute ventilation, and end-tidal PCO_2_ were all significantly higher at baseline in the session after food intake than in the session before food intake. On the other hand, there was no significant difference in chemosensitivity to CO_2_ between the sessions before and after food intake (1.60 ± 0.62 vs. 1.53 ± 0.62 l min^−1^ mmHg^−1^).

**Conclusions:**

Food intake does not influence respiratory chemosensitivity to CO_2_ in young adults, which is different from infants. This suggests that control of respiration differs between young adults and infants and that the elevated minute ventilation after food intake in young adults is not caused by a change in respiratory chemosensitivity.

## Background

It is well known that food intake influences several physiological parameters. For example, food intake increases metabolism and body temperature [1–3]. This is the so-called postprandial thermogenesis or thermic effect of food. We recently reported that food intake also influences the respiratory response at rest [1]—i.e., food intake increases minute ventilation (*V*_*E*_) in resting young individuals. In that study, we speculated that this elevation in *V*_*E*_ was caused by an elevation in the H^+^ ion concentration related to increases in CO_2_ output (*V*CO_2_) and the plasma noradrenaline concentration [4], which can be induced by an increase in sympathetic nervous system activity [5]. Young adults in that study exercised at 50% of peak oxygen uptake 90 min after food intake, and the increase in metabolism elicited by the food intake was still present during the subsequent exercise. Interestingly, we found that although *V*CO_2_ and end-tidal PCO_2_ (P_ET_CO_2_) were increased by food intake during exercise, *V*_*E*_ was not [1]. On the other hand, when we previously compared the ventilatory responses during exercise with participants breathing CO_2_-enriched air or room air, we found that breathing CO_2_-enriched air elevated PCO_2_ and *V*_*E*_ [6]. We have thus obtained differing results regarding the impact of CO_2_ on *V*_*E*_.

It has also been reported that respiratory chemosensitivity is subject to alteration by several factors. For example, it was reported that exercise (at 19%, 26%, and 34% of maximal oxygen uptake) increased respiratory chemosensitivity to CO_2_ relative to the resting state [7] and that a rise in body temperature of > 0.7 °C increased respiratory chemosensitivity to CO_2_ [8]. From these findings, it is plausible that respiratory chemosensitivity to CO_2_ is increased during exercise after food intake. However, our previous study [1] did not reveal higher *V*_*E*_ during exercise after food intake. From that finding, we hypothesized that *V*_*E*_ remains steady as a result of redundant compensatory mechanisms [1, 9]. On the other hand, Durand et al. [10] tested the effect of feeding on respiratory responses in the newborn infant and reported that feeding suppresses respiratory chemosensitivity to CO_2_. It is therefore possible that food intake suppresses respiratory chemosensitivity to CO_2_ in infants, though it remains unclear whether food intake changes respiratory chemosensitivity in adults. To address this issue, we compared respiratory chemosensitivity to CO_2_ measured before and after food intake in young adults.

## Material and methods

### Participants

Eleven healthy participants [four males and seven females, mean age = 21 ± 2 (SD) year; height = 169.0 ± 7.4 cm; weight = 59.7 ± 8.5 kg] participated in the study. None of the participants were smokers, and none were taking any medication. All of the female participants were studied within 10 days of menstruation, and none were taking oral contraceptives, which contain female hormones. The study was approved by the Research Ethics Committee of the University of Shizuoka and conformed to the provisions of the Declaration of Helsinki. Written informed consent was obtained from all participants.

### Test meal

Test meals were designed for each participant. After estimating the basal metabolic rate for each subject using the equation of Ganpule et al. [11], we estimated the total energy expenditure by multiplying the estimated basal metabolic rate by the estimated average physical activity level [12]. For six of the participants, that factor was 1.75; for the remaining five, it was 2. The amounts of carbohydrate, protein, and fat in the meals were based on the Dietary Reference Intakes for the Japanese [13]. As a result, test meals contained 117.6 ± 17.4 g of carbohydrate, 30.9 ± 8.8 g of protein, and 20.2 ± 5.4 g of fat. The total energy content was 3332 ± 633 kJ.

### Experimental design

The experiment was conducted in the morning, and the participants were all asked to abstain from strenuous exercise and not to consume any food or alcohol during the 12 h prior to the experiment. After each subject came to the laboratory, they voided urine, were weighed, and sat in a chair to rest for 30 min. During this period, a heart rate (HR) monitor (S810i, Polar, Finland) was attached. HR was recorded every 5 s during the experiment and was averaged over 30-s periods. Just before the measurements were begun, sublingual temperature (*T*_sl_) data were collected via copper-constantan thermocouples, which were sampled every 1 s using a data logger system (WE7000, Yokogawa, Japan) and averaged over 30-s periods. A mass-flow sensor and a gas-sampling tube were then connected to the mask, after which respiratory chemosensitivity to CO_2_ was measured using a rebreathing method [14, 15]. Participants consumed test meals after the measurement. *T*_sl_ and respiratory chemosensitivity were then measured 90 min after eating using the same procedure. The experiments were carried out in an experimental laboratory maintained at 25 °C and 40–60% relative humidity.

### Rebreathing test

The participants wore a mask connected to a closed one-way circuit with a 6-l rubber bag containing the test gas (7% CO_2_, 43% O_2_, 50% N_2_). For the first 5 min with the stopcock open (“the one-way rebreathing circuit”), the participants breathed room air to measure control ventilation values. The stopcock was then closed, and rebreathing was begun. Rebreathing was terminated when the inspired CO_2_ fraction reached 9.2%. Rebreathing time ranged from 4 to 6 min in each participant. Respiratory parameters and gas concentrations were monitored breath-to-breath (AE-310S, Minato Medical Science, Japan). This test was performed twice with a 20-min recovery period between tests, and the mean values were evaluated for respiratory chemosensitivity, which was calculated as the slope of the regression line relating P_ET_CO_2_ to *V*_*E*_. Because a ventilatory recruitment threshold was observed at around P_ET_CO_2_ = 45 mmHg in several participants, regression analyses were conducted with data for P_ET_CO_2_ > 50 mmHg. Previous studies showed that there is no significant difference in respiratory chemosensitivity to CO_2_ between steady-state and rebreathing methods when they are used under normal acid-base conditions [16, 17]. Therefore, the present results are comparable to results obtained using steady-state methods.

### Statistical analysis

All values are reported as means ± SD. Paired *t* tests were used to compare the “before food intake” and “after food intake” sessions among the measured variables. Values of *P* < 0.05 were considered significant.

## Results

Baseline *T*_sl_, HR, oxygen uptake (*V*O_2_), *V*CO_2_, *V*_*E*_, respiratory frequency (*f*_R_), and P_ET_CO_2_ were all significantly higher during the session after food intake (Table 1). The baseline respiratory exchange ratio, tidal volume (*V*_*T*_), and end-tidal PO_2_ did not significantly differ between sessions.

**Table 1:** Baseline levels of the measured parameters

	Before food intake	After food intake
*T*_sl_, °C	36.1 ± 0.3	36.4 ± 0.3*
HR, beats/min	62 ± 8	65 ± 7*
*V*O_2_, ml/min	240 ± 27	270 ± 39*
*V*CO_2_, ml/min	202 ± 60	233 ± 70*
RER, unit	0.84 ± 0.22	0.86 ± 0.22
*V*_*E*_, l/min	9.7 ± 3.3	11.0 ± 3.4*
*V*_*T*_, ml	682 ± 154	681 ± 167
*f*_R_, breaths/min	15 ± 4	17 ± 3*
P_ET_O_2_, mmHg	100 ± 8	101 ± 8
P_ET_CO_2_, mmHg	40 ± 3	41 ± 3*

Values are means ± SD

*RER* respiratory exchange ratio, *P*_*ET*_*O*_*2*_ end-tidal PO_2_

*P < 0.05 before vs. after food intake

**Fig. 1:**
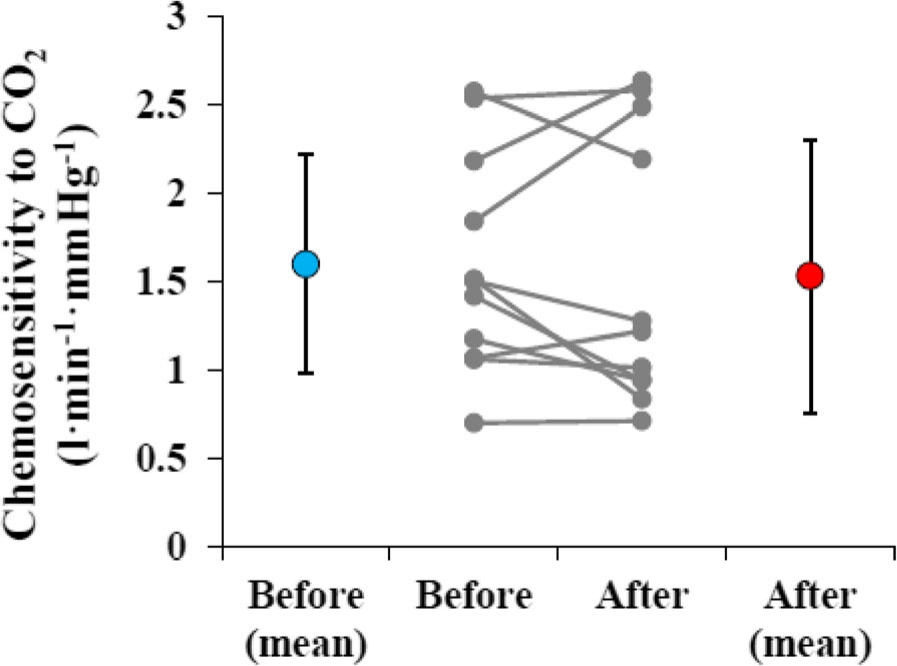
Mean (± SD) respiratory chemosensitivity to CO_2_ before (blue circle) and after (red circle) food intake. Gray circles and lines depict individual data

Figure 1 shows the respiratory chemosensitivity to CO_2_. There was no significant between-session difference in respiratory chemosensitivity to CO_2_ (1.60 ± 0.62 l min^−1^ mmHg^−1^ in the before food intake session vs. 1.53 ± 0.77 l min^−1^ mmHg^−1^ in the after food intake session, *P* = 0.59, effect size *d* = 0.13). However, six of the 11 participants showed higher chemosensitivity before food intake.

## Conclusions

The present study showed that food intake leads to increases in *T*_sl_, *V*O_2_, *V*CO_2_, *V*_*E*_, and P_ET_CO_2_, which is consistent with earlier studies [1–3]. Moreover, the results suggest that the elevated *V*_*E*_ is not caused by a change in respiratory chemosensitivity, and they support our earlier speculation that the elevation in *V*_*E*_ is caused by increases in the H^+^ ion concentration and plasma noradrenaline concentration [1].

Our results showed that food intake did not influence respiratory chemosensitivity to CO_2_, which is in contrast to an earlier report [10]. It may be that this discrepancy reflects the difference in the type of food eaten by the participants. The infants who participated in the earlier study took in milk, while the young adults participating in the present study ate test meals containing rice, chicken, and other things. To test this idea, it will be necessary to examine the effect of different food types on respiration. Furthermore, food intake elicited an elevation in *T*_sl_ of only 0.3 °C in the present study. It is likely that no change in respiratory chemosensitivity can be explained by this small elevation in *T*_sl_. Nonetheless, in infants, respiratory chemosensitivity to CO_2_ was changed by feeding, which suggests this change cannot be explained simply based on body temperature. To understand this discrepancy, it will be necessary to clarify the differences relating to respiration between young adults and infants.

González-Castillo et al. [18] compared the changes in gene expression that occur in the ventral respiratory column of the ventrolateral medulla, which is involved in determining respiratory rhythm and central chemoreception, between adult and neonatal rats. Their analysis confirmed the differential expression of 84 genes involved in the expression of neuronal ion (K^+^, Na^+^, and Ca^2+^) channels. Although it is not clear how these genes relate to respiratory chemosensitivity to CO_2_, it may be that the differences their expression relate to the different responses to CO_2_ in neonates and adults. Further study will be necessary to clarify the differences in respiratory control and to determine how respiratory chemosensitivity changes with aging.

In summary, our results suggest that food intake does not suppress respiratory chemosensitivity to CO_2_ in young adults, which is different from the situation in infants and suggests that respiratory chemosensitivity does not directly influence ventilatory responses after food intake. These observations do not support the hypothesis that blunted respiratory chemosensitivity to CO_2_ underlies the attenuation of the increase in *V*_*E*_ during exercise after food intake. On the other hand, these results support the hypothesis that *V*_*E*_ remains steady during exercise after food intake as a result of redundant mechanisms.

## Data Availability

The datasets used and/or analyzed during the current study are available from the corresponding authors on reasonable request.

## Abbreviations

*f*_R_: Respiratory frequency
HR: Heart rate
P_ET_CO_2_: End-tidal PCO_2_
*T*_sl_: Sublingual temperature
*V*CO_2_: CO_2_ output
*V*_*E*_: Minute ventilation
*V*O_2_: Oxygen uptake *V*_*T*_ Tidal volume
